# Domestic Summary

**Published:** 1839-10-12

**Authors:** 


### DOMESTIC SUMMARY.

### MEDICAL CONVENTION.

The following gentlemen have been elected Delegates to the Medical Convention for revising the Pharmacopœia of the United States, to meet at Washington on the first Wednesday of January, 1840, viz.:

*By the College of Physicians of Philadelphia*—George B. Wood, M. D., Franklin Bache, M. D., Henry Bond, M. D.

*By the Medical Society of the District of Columbia*—Thomas Sewall, M. D., James C. Hall, M. D., Nicholas W. Worthington, M. D.

*By the New Jersey Medical Society*—Lewis Condict, M. D., —— Forman, M. D.

*By the Albany Medical College*—David M. McLachlan, M. D.

*By the Rhode Island Medical Society*—Theophilus C. Dunn, M. D., Usher Parsons, M. D.

*By the New Hampshire Medical Society*—Dixi Crosby, M. D., Daniel D. Adams, M. D., Amos Twitchell, M. D.; and as substitutes, John B. Dousman, M. D., Josiah Bartlett, M. D., Robert Burns, M. D.

*By the Medical Society of Delaware*—William M. Morris, M. D., Cuthbert S. Green, M. D., James Couper, M. D.

*By the College of Physicians and Surgeons of Lexington, Kentucky*—John C. Richardson, M.D., John C. Darby, M. D., Caleb M. Cloud, M. D.

Published by direction of the Medical Convention, which met at Washington, in January, 1830.

LEWIS CONDICT, M. D., President.

*New Method of Treatment for Un-united Fracture.* Dr. J. S. Heard, late of the New York Hospital, reports, in the New York Medical Journal, several cases of un-united fracture, successfully treated according to a suggestion of Dr. J. Kearney Rodgers. It consists in *removing the extremities of the fractured bone and connecting the fragments by silver-wire.* This operation has not been adopted in any other place than the New York Hospital. Four cases are reported, in which the operation was successful. We subjoin the history of a case, illustrating the treatment:

"C. G., aged 26, a German, was admitted into the New York Hospital, September 28th, 1838, with a simple fracture of the radius of the right fore-arm, about two inches above the wrist-joint. The injury was caused by his hand being caught in machinery, and bent forcibly back. There was considerable contusion of the whole forearm, and some laceration of the hand. The fracture of the radius was very oblique, and the lower end of the upper fragment projected nearly through the integuments. The fracture was reduced, and the patient being placed in bed, the arm was laid in a flexed position on a pillow, covered with oil silk, and lot. evap. applied. As soon as the state of the arm would allow, the requisite pads and splints were applied, and the arm supported in a sling. At the expiration of 4 weeks from the application of the splints, they were removed, but no union had taken place. It was now suggested that a strip of muscle interposed between the fragments, was the cause of non-union.

A splint, with a hand piece setting off from the body of the splint at an angle of about 110°, was now applied upon the palmar side of the forearm, in order to elevate the lower fragment. The interosseous pads being only applied between the ulna and this lower fragment, the usual splint was placed on the dorsal side. The arm was thus supported in a sling for six weeks; at the expiration of which period, upon removing the apparatus, there was still no union. The same apparatus was replaced and the patient put under the use of mercury, but it produced profuse diuresis and was discontinued. The splints were allowed to remain applied until January 10th, when Dr. Rodgers made an incision about 2½ inches in length, over the seat of fracture on the palmar side of the fore-arm; upon exposing the fragments, the radial artery was found running between them; the lower fragment was drawn down to the ulna, while the upper one was separated near an inch from it, a slip of the extensor carpi radialis muscle intervening. The radial artery was divided and secured at both extremities, the intervening muscle was severed, and the ends of the bone removed with the bone pliers. A hole was now drilled into each extremity, and a silver wire being passed through, the bones were approximated by twisting the wire, the end of which was allowed to hang from the wound. The edges of the wound being brought together by adhesive straps, the ordinary pads and splints were applied, and the arm suspended in a sling. A slight attack of erysipelas occurring, required the confinement of the patient to bed, and the removal of the splints; upon the subsidence of the erysipelas in a few days, the splints were re-applied.

*February 2d.* The wire came away to-day, the loop being entire. The splints and bandages were continued until April 2d, when, upon removing them, the union was found to be perfect. He remained in the hospital, using passive motion, until April 15th."

*A Case of Dislocation of the Patella upon its Axis, with Comments.* By John Watson, M.D.—The following case is of some importance, inasmuch as it establishes a new fact, or, to say the least, a controverted point in the surgical pathology of the patella.

Henry Burton, a carman, of rather slender frame, and about thirty-five years of age, was on horseback in a crowd at the Park, August 21st, 1839, waiting to see the public reception of the Hon. Henry Clay. In the confusion of the crowd, another horseman backed his horse against him, and the animal's hip striking against Burton's right leg, injured him so severely that he was unable to say how, or in what direction, he received the shock. He was carried immediately to a neighbouring hotel.

I saw him soon after the accident, in connection with Dr. L. C. Ferris, Dr. Stearns of the northern part of the city, and Dr. Thomas F. Cock. The patient was complaining of severe pain; the leg was perfectly straight, but could be flexed about to an angle of 140° without increasing his suffering. The patella appeared to be slightly drawn up, and it was twisted upon its axis, presenting its outer edge, in a prominent hard line, in front of the knee; its inner edge, resting either in the groove between the condyles of the femur, upon which its posterior face should naturally play, or in the small indenture on the anterior face of the femur, immediately above this groove. The anterior surface of the patella was turned inward, its posterior surface outward, and it rested nearly at right angles with its natural position. Its upper and lower attachments were both preserved, and could be distinctly felt; and a sort of band appeared to pass from its under, or, as it now lay, its outer face, inward to the deeper portion of the knee-joint. This band, as I conceived, was caused, either by the tension of the capsular ligament, or by the rupture of its edge, as it passes from the outer side of the patella. The position of the bone was so well marked that no one at all acquainted with the anatomy of the part, could mistake the nature of the accident.

With the leg extended, and the anterior muscles of the thigh forced downwards as much as possible, pressure was made upon the patella, with the expectation of forcing down its prominent edge, and pushing the bone directly into its proper position. The effort was followed only by a severe increase of pain; the bone remained permanently fixed. Another attempt was made to cant its posterior edge inward, and to bring its anterior edge outward, without pressing it against the condyles of the femur, by forcing the head of a key against the posterior, now the outer face of the patella, (using this as a fulcrum,) and pressing the prominent edge of the bone towards the outer condyle. This manœuvre gave him no pain; but was as fruitless in its result as the other. At length the knee was forcibly bent, and immediately straightened again; and then, by canting the patella as before, and pushing it slightly downwards and inwards, it sprung with a sudden snap into its proper position. The reduction would, doubtless, have been facilitated by flexing the thigh strongly upon the pelvis. This, however, was not attempted, and the manipulation, just described, answered every purpose.

As soon as the bone was replaced the pain ceased; and the patient thinking himself well again, stated that he could now walk as well as ever. A straight splint was applied behind the limb, so as to secure the joint, and the patient was sent home in a carriage, with directions to keep the knee at rest for a few days, and to apply to it an evaporating lotion.

Most authors, who have spoken of luxations of the patella, state that this bone is subject only to the outward and the inward lateral displacement. Sir Astley Cooper admits, also, an upward, and Boyer speaks of both an upward and a downward displacement. But the two former displacements, according to all the writers who have hitherto dwelt upon these accidents, are the only ones that can occur, independent of a rupture, either of the ligament of the patella, or of the common tendon of the extensor muscles of the leg. In a hasty perusal of most of the surgical authorities, as well English as French, I have met with but one instance bearing any relation to the case just related. "I was informed by Mr. Welling, formerly surgeon at Hastings," says Sir Astley Cooper, "that he was called to a case in which the patella was dislocated upon its edge. The nature of the accident was very obvious, as the edge forced up the integuments to a considerable height between the condyles on the fore-part of the joint. Mr. Welling reduced the dislocation, but with considerable difficulty, by pressing the edges of the bone in opposite directions."

Sir Astley Cooper gives this observation in a distinct paragraph, without remarks upon it; and as if in need of confirmation, he allows it no place in his enumeration of the several luxations of the patella. Nothing of the sort is mentioned in Mr. S. Cooper's Dictionary. Boyer, after speaking of the lateral displacements, states, that independent of these, some surgeons have thought that this bone might be luxated "by turning half-way round upon itself, and resting with its edge in the articular pulley of the femur; but," says he, "it is impossible to conceive how the tendon of the extensor muscles of the limb, and the ligament of the patella, could be twisted in unison with the rotation of the bone upon itself; and it is still less conceivable how these parts could be completely turned round from before backwards, as some pretend to have seen."

Where the luxation of the patella outward is but partial, the signs of the accident must be more like those of the case I have described, than where the outward luxation is complete. Yet the difference is still too striking to allow this accident to be confounded with a partial luxation outward. "The external displacement," says M. Sanson, "is easily recognised by pain, inability to walk, and deformity of the knee; by the ease with which the inner edge of the articular pulley of the femur, as well as that part of the surface of this pulley, which is not covered by the patella, can be felt beneath the integuments; by the prominence upon the external edge of this pulley, formed by the patella, the outer edge of which is *slightly directed forward*, whilst its anterior face is turned *a little inward*, and its posterior face is so situated on the external condyle, that the outer portion of it may be easily touched; by the tension and external deviation of the tendon of the muscles on the anterior part of the thigh, and of the inferior ligament of the patella; by the permanent extension of the leg."

But in the case which I have described, the leg could be slightly flexed; no part of the pulley, except its elevated borders at the condyles of the femur, could be felt; *the patella was drawn upwards, and twisted nearly at right angles with its proper position, so that its anterior face was directed inwards, and its outer edge was thrown completely forward, forming an uneven and very prominent line beneath the skin in front of the joint.*

But though the position of the patella in this case was very different from that which it occupies in the subluxation outwards, yet it is easy to see how the latter accident might be resolved into this. For the patella in slipping outward, while passing over the outer edge of the pulley of the femur, must, from the very shape of the bones, have its outer edge tilted forward, and its inner edge, in consequence, thrown a trifle backward. Now, whilst in this position, and before the patella is allowed to reach fairly to the anterior face of the condyle, a sudden contraction of the rectus and vasti muscles must tend to bring the bone back again within the line of their action: so that instead of slipping completely over the condyle, it will either fall back into its natural position, or its inner edge being arrested, and the bone being thus prevented from moving inwards bodily, its outer edge must be drawn forward toward the axis of the limb, and the accident I have described will be thus produced.

Though some authors describe the lateral displacement as one, occasionally difficult of reduction, yet as a general rule, the bone slips into its place very readily, from the slightest pressure upon it, especially where the knee is extended, and the thigh flexed upon the pelvis; or, as some direct, by attempting to flex the knee, and thus elongate the muscles in front of the thigh. But in the case of Burton, just related, as well as in the case reported by Mr. Welling to Sir Astley Cooper, the patella was firmly fixed, and reduced only after considerable manipulation.

In a case of complete dislocation of the patella outward, which occurred to me, July 25th, 1838, the slightest push of the thumb was sufficient to restore the bone to its proper place. And somewhat contrary to the diagnostic signs usually given as characteristic of this accident, the leg in this instance was slightly but firmly flexed.

The case was that of a young lady of about 22 years of age, remarkably fleshy, but of lax fibre. The accident occurred in dancing. In working the joint immediately after reducing the dislocation, it gave a rough and rubbing sound, that might have been mistaken for the crepitus of a fractured bone. Some œdema and effusion within the capsule followed the accident. But the application of leeches, rest in an elevated position, evaporating lotions, and finally a roller, were sufficient, in the course of a week or ten days, to restore the joint to its proper condition.

Authors differ as to the frequency of simple dislocations of the patella. By referring to some, we might be led to believe these accidents of very frequent occurrence. Such, I suspect, is not the fact. Although I have heard of a few cases in the practice of my acquaintances, the two which I have now mentioned are all that have fallen immediately under my own observation. Mr. Liston, in his Elements of Surgery, states that he has never met with a single case.

*N. Y. Med. and Surg. Journ.*

